# Time-course transcriptomic analysis of *Petunia* ×*hybrida* leaves under water deficit stress using RNA sequencing

**DOI:** 10.1371/journal.pone.0250284

**Published:** 2021-04-26

**Authors:** Suejin Park, Asela J. Wijeratne, Youyoun Moon, Nicole L. Waterland

**Affiliations:** 1 Division of Plant and Soil Sciences, West Virginia University, Morgantown, West Virginia, United States of America; 2 Department of Biological Sciences, Arkansas State University, Jonesboro, Arkansas, United States of America; Universidade de Lisboa Instituto Superior de Agronomia, PORTUGAL

## Abstract

Water deficit limits plant growth and development, resulting in quality loss of horticultural crops. However, there is limited information on gene regulation and signaling pathways related to water deficit stress response at multiple time points. The objective of this research was to investigate global gene expression patterns under water deficit stress to provide an insight into how petunia (*Petunia* ×*hybrida* ‘Mitchell Diploid’) responded in the process of stress. Nine-week-old petunias were irrigated daily or placed under water stress by withholding water. Stressed plants reduced stomatal conductance after five days of water deficit, indicating they perceived stress and initiated stress response mechanisms. To analyze transcriptomic changes at the early stage of water deficit, leaf tissue samples were collected 1, 3, and 5 days after water was withheld for RNA sequencing. Under water deficit stress, 154, 3611, and 980 genes were upregulated and 41, 2806, and 253 genes were downregulated on day 1, 3, and 5, respectively. Gene Ontology analysis revealed that redox homeostasis processes through sulfur and glutathione metabolism pathways, and hormone signal transduction, especially abscisic acid and ethylene, were enriched under water deficit stress. Thirty-four transcription factor families were identified, including members of AP2/ERF, NAC, MYB-related, C2H2, and bZIP families, and TFs in AP2/ERF family was the most abundant in petunia. Interestingly, only one member of GRFs was upregulated on day 1, while most of TFs were differentially expressed on day 3 and/or 5. The transcriptome data from this research will provide valuable molecular resources for understanding the early stages of water stress-responsive networks as well as engineering petunia with enhanced water stress tolerance.

## Introduction

Faced with water scarcity, drought is one of the critical issues in the world. Climate change is predicted to cause more severe droughts in the future, aggravating the shortage of water resources. As water supplies are not able to meet water demands, regulation of water use becomes inevitable in agriculture. Water deficiency is a major constraint to crop productivity worldwide, thus leading to substantial crop loss and ultimately presenting issues of food insecurity [1].

Ornamental plants have become an important portion in the horticultural industry. A variety of ornamental plants are widely used in home gardening, landscaping, and the cut flower industry [2]. The wholesale value of floriculture is estimated to be more than $4.63 billion, and bedding plants make up about 47% of the total value of the ornamental plant market for 2018 in United States [2]. Ornamental plants are produced at a premium quality, but their quality often declines during shipping and retailing without appropriate temperatures and irrigation. These adverse environments accelerate substrate drying and plant wilting. Crop losses due to these poor postproduction conditions are estimated to be 5% to 20% of unsalable crops [3]. Therefore, the development of cultivars tolerant to water stress are needed to minimize the decline of crop quality and crop loss during shipping, retailing, and the loss of crops at the final destinations. Better understanding of response mechanisms to water stress would assist in improving water stress tolerance in ornamental crops.

Plants have a wide range of morphological, physiological, and biochemical processes to cope with water deficit stress. These processes include the development of large and deep root systems, stomatal closure, accumulation of osmolytes, increase of antioxidant activity, and changes in metabolism and signaling of phytohormones [1]. Various genes are involved in these stress responses, suggesting water deficit stress tolerance is mediated by more complex mechanisms than previously thought [4]. It has been suggested that stress-responsive genes can be classified into two groups [5]. The first group of genes plays a direct role in stress tolerance, including enzymes involved in osmotic adjustment, antioxidant proteins, ion transporters, detoxification enzymes, chaperones, late embryogenesis abundant proteins, and various proteases [5]. The second group includes the regulatory genes encoding protein kinases, protein phosphatases, and transcription factors (TFs) that mediate signal transduction pathways and transcription of downstream target genes [5].

Despite considerable efforts to understand the water stress response pathways, comprehensive information on genetic regulatory networks and tolerance under water deficit still remain elusive. While Arabidopsis has been extensively investigated, it cannot represent all other extant species [6]. Furthermore, it has been suggested that molecular mechanisms of water stress response may differ between Arabidopsis and other plants, although some water deficit stress response pathways may be conserved [4]. Therefore, it is important to investigate the crop of interest so that molecular mechanisms specific to that plant may be identified.

With rapid advances in next-generation sequencing, RNA sequencing (RNA-seq) has broadened the scope of gene expression studies from a few genes to the whole genome and from a limited number of model plants to a vast range of non-model species. While microarray requires previous genome annotation and pre-synthesized nucleotide probes, RNA-seq can be used to analyze the transcriptome of organisms in the absence of a reference genome and to discover novel genes [7]. Additionally, RNA-seq can provide more accurate qualitative and quantitative information of gene expression than the microarray-based assays [8]. Because of these advantages, RNA-seq has been widely used to sequence transcripts and profile global gene expression data in a variety of plant species under abiotic and biotic stresses. However, to the best of our knowledge, no research has been performed on the transcriptomic profiling under water deficit in petunia using RNA-seq.

Petunia (*P*. ×*hybrida*) was investigated in this study because it is one of the most important ornamental crops used worldwide in landscaping. In addition, the genus Petunia is a member of the Solanaceae family along with tobacco, tomato, and pepper, and it represents a more diverse range of plants than Arabidopsis [6]. For several decades, petunia research had focused on flavonoid synthesis, floral development and senescence, but few studies regarding abiotic stress response at the molecular level have been conducted. Two zinc finger TFs (ZPT2-2 and ZPT2-3) have been studied for their roles in dehydration, wounding, and cold [9]. However, it is expected that various genes are involved in abiotic stress responses. In order to improve water deficit tolerance in petunia, it is desired to understand genome-wide expression profiling in responding to water deficit.

In this study, RNA-seq was performed to generate time-course transcriptome data with petunia. The specific research objectives were to 1) perform and analyze transcriptome to provide a global gene expression profile at three time points of water deficit, 2) identify differentially expressed genes (DEGs), and 3) determine specific biological processes involved in water deficit stress, and 4) identify TFs associated with water stress tolerance in petunia. The time-course transcriptomic data obtained from this project will provide valuable insight into molecular mechanisms underlying the early stage of water deficit stress responses, and can be utilized to generate plants with enhanced water stress tolerant traits through breeding and genetic engineering.

## Materials and methods

### Plant material, water deficit treatment, and tissue collection

Seeds of *P*. ×*hybrida* ‘Mitchell Diploid’ were sown in a 288-plug tray. Seedlings were transplanted into 11-cm pots with soilless media (Sunshine^®^ Mix #1; Sun Gro Horticulture, Agawam, MA, USA)(one plant per pot). Plants were grown under natural irradiation with supplemental lighting provided as needed by high-pressure sodium lighting (600W HS200 deep reflector, Hortilux, Pijnacker, The Netherlands) in a greenhouse (Morgantown, WV, USA). Plants were fertilized with Peter^®^ Excel Cal-Mag 15N-2.2P-12.5K (Everris, Marysville, OH, USA) at 200 mg N·L^-1^, which was reduced to 100 mg N·L^-1^ one week before water deficit treatment. Nine-week-old petunias were randomly divided into two groups: control (C) and water-stressed (S). Plants in the control group were irrigated daily with 100 mg N·L^-1^ until the end of the experiment, while the water deficit stress group received no water during water treatment for up to five days. Stomatal conductance was measured daily, and leaf tissues were collected on the 1st, 3^rd^, and 5th day of water treatment with three replications (or three plants, n = 3). A fully expanded leaf (the seventh or eighth leaf from the top) per plant was collected. Each treatment had nine individual plants, and three plants were randomly selected for each time point. Instead of using control tissues collected at one time, control samples were collected together with stressed plants at the corresponding time to minimize any circadian and/or environmental effect at each time point.

### Measurement of stomatal conductance

Prior to leaf collection, stomatal conductance of three leaves per plant was measured between 10 am to 2 pm with a portable photosynthesis system (LI-6400XT; LI-COR Inc., Lincoln, NE, USA). A leaf was clamped into an extended chamber with clear top and bottom covers (Extended Reach 1 cm Chamber LI6400-15; LI-COR). Environmental conditions in the chamber were set at 400 μmol·m^-2^·s^-1^ CO_2_, and 25°C. Readings were conducted from 1000 to 1400 HR. Data of stomatal conductance are the means of measurements from three plants (n = 3). Reduced stomatal conductance is an indicator of plant response to water deficit stress. Analysis of variance (ANOVA) was performed by SAS 9.3 (SAS Institute, Inc., Cary, NC).

### Total RNA extraction, RNA-seq library construction, and sequencing

For RNA-seq analysis, leaf samples were collected one, three, and five days after withholding water from control and water-stressed plants, named herein C1, C3, C5, S1, S3, and S5, respectively. Three biological replicates were used (n = 3). Total RNA was extracted using Trizol Reagent (Invitrogen, USA), and RNA was purified by phenol/chloroform extraction following the standard procedures. Total RNA quality was evaluated by an Agilent 2100 Bioanalyzer (Agilent, Santa Clara, CA, USA), with the average RNA integrity number of 7.54. cDNA libraries for sequencing were prepared using the TrueSeq RNA Sample Prep Kit according to the manufacturer’s instructions (Illumina Inc., San Diego, CA, USA) at the WVU Genomics Core Facility. These libraries were sequenced on an Illumina HiSeq1500 (Illumina Inc., San Diego, CA, USA) at Marshall University Genomics Core Facility, and reads were generated in 50bp paired-end format.

### Data processing, *de novo* assembly, and annotation

Raw reads in FASTQ format were subjected to sequence quality control using FastQC (http://www.bioinformatics.babraham.ac.uk/projects/fastqc). Trimmomatic (http://www.usadellab.org/cms/?page = trimmomatic)(version 0.39) was used to remove adapters and filter/trim the low-quality score reads if Phred quality is less than 25. Trimmed reads with minimum length of 40 bp were used for *de novo* assembly using Trinity (version 2.2.0) [10]. *De novo* assembly was used since a reference genome for *P*. *×hybrida* was not available. After removing contigs shorter than 200 bp, CD-HIT-EST [11] was used to cluster sequences with 90% identical contigs/isoforms to obtain nonredundant transcripts. The completeness of the *de novo* assembly was evaluated using Benchmarking Universal Single-Copy Orthologs (BUSCO) against eudicot sequences [12]. To perform functional annotation, all transcripts were compared to *Solanum lycopersicum* protein database (ftp://ftp.ncbi.nlm.nih.gov/genomes/Solanum_lycopersicum/protein/)(ITAG version 3.1) with cut-off E-value of 1 × 10^−5^ using BLASTx [13]. The resulting BLAST results were fed into Blast2GO (http://www.blast2go.com) [14] to predict Gene Ontology (GO) annotation, describing biological processes, molecular functions, and cellular component. Web Gene Ontology Annotation Plot (WEGO) was used to visualize GO categories [15].

### Gene expression quantification and differential expression analysis

The RNA-seq reads were aligned to the assembled transcripts using Bowtie2 (http://bowtie-bio.sourceforge.net/bowtie2) [16], and transcript abundance was assessed by RNA-seq By Expectation Maximization (RSEM) software (http://deweylab.biostat.wisc.edu/resm) [17] bundled with the Trinity package [10]. The expression levels were normalized with Trimmed Mean of M values (TMM). Differentially expressed genes were identified if a false discovery rate (FDR) < 0.05 and a two-fold or greater change in transcript levels between control (C) and stress (S) treatment at each time point (C1 vs. S1, C3 vs. S3, and C5 vs. S5) using edgeR Bioconductor (http://www.bioconductor.org) [18]. To identify unique and common genes across the three time points, Venn diagrams were generated by VENNY 2.1 (http://bioinfogp.cnb.csic.es/tools/venny/). Gene Ontology enrichment analysis was further performed by Fisher’s Exact Test (FDR < 0.05) using Blast2GO [14]. Kyoto Encyclopedia of Genes and Genomes (KEGG) [19] was used to characterize associated pathways with a threshold bit-score value of 60 (default). Among DEGs, the list of TFs was obtained by using Plant Transcription Factor Database (PlantTFDB, http://planttfdb.cbi.pku.edu.cn) [20].

### Validation of differentially expressed genes using quantitative PCR

Five genes with distinct expression changes over three time points were selected to verify the accuracy of RNA-seq data. Expression levels of the selected genes in water-stressed leaves were analyzed by quantitative PCR (qPCR), while the *elongation factor 1α* (*PhEF1α*) from petunia was used as an internal control [21]. Reverse transcription was performed using qScript^TM^ cDNA Synthesis Kit (Quanta Biosciences, Gaithersburg, MD, USA) according to the manufacturer’s protocol. The qPCR was performed by QuantStudio^TM^ 3 Real-Time PCR System (Thermo Fisher Scientific, Waltham, MA, USA). The PCR reaction mixture contained 10 ng cDNA template, 5 μl PowerUp^TM^ SYBR^TM^ Green Mater Mix (Thermo Fisher Scientific, Waltham, MA, USA), 0.5 μl forward and reverse primers (10μM), and water up to 10μl. The amplification conditions were as follows: an initial incubation at 50°C for 2 m and 95°C for 2 m, followed by 40 cycles of 95°C for 15 s, 60°C for 15 s, and 72°C for 1 m. A melting curve (55–95°C with a heating rate of 0.15°C‧s^-1^ and a continuous fluorescence measurement) was performed for each qPCR reaction. The relative expression of each target gene in one sample was calculated as follows: 2^-(Ct target gene—Ct *PhEF1α*)^. Primers of the internal control and target genes listed in (S1 Table), and the PCR product size from each amplicon ranged from 100 to 110 bp. All the samples were measured in three technical replications.

## Results

### Changes in stomatal conductance under water deficit stress

**Fig 1:**
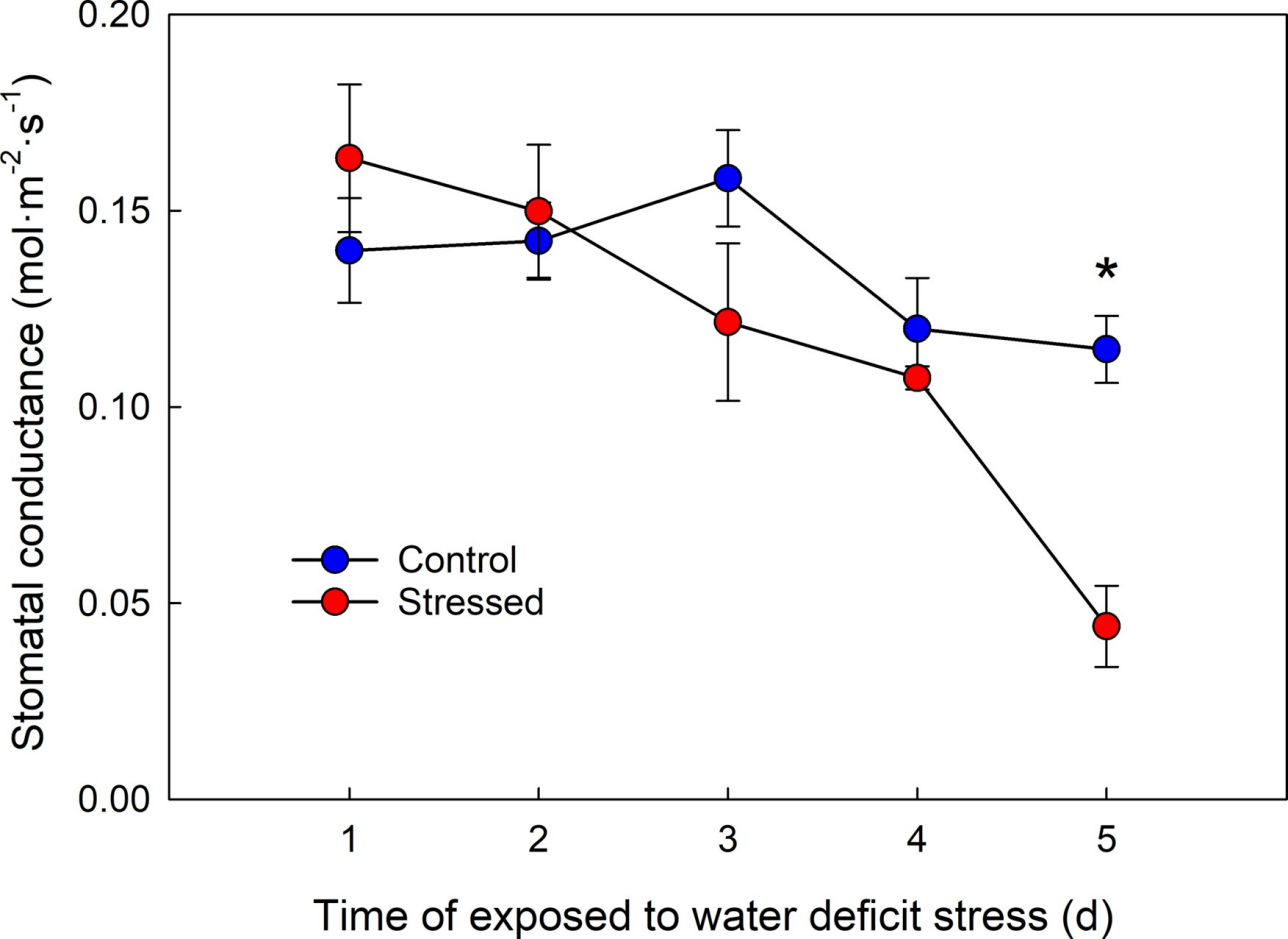
Stomatal conductance of control and water-stressed plants for five days after withholding water. Vertical bars are standard errors of the means with three replications (n = 3). * Significant at *P* ≤ 0.05.

To determine the duration of water deficit stress treatment, stomatal conductance was measured daily, because stomatal closure is a sign of perception of the stress. Five days after withholding water, stressed plants showed decreased stomatal conductance by 38% compared to control plants (*P* = 0.0348, Fig 1). No visual changes were not observed on day 5, such as wilting or discolored leaves (data not shown). Leaf samples were collected on day 1, 3, and 5 from well-watered (control) and water-stressed plants with three replications. Total 18 libraries (two treatments × three time points × three replications libraries were used for RNA-seq analysis.

### Transcriptome sequencing and *de novo* assembly

Nearly 264 million paired-end reads of 50 bp were generated from 18 libraries, ranging from 8,898,370 to 18,716,488 reads per library, with a mean of 14,631,356 (S2 Table). After preprocessing and quality trimming, more than 262 million reads were obtained with the total nucleotides of 13.1 giga bases, approximately 9.3-fold coverage of the estimated genome size of parental species of *P*. ×*hybrida* [22]. *De novo* assembly using Trinity generated 76,601 contigs with the total contig length of 63,383,628 bp and average contig length of 827 bp (Table 1). In order to remove redundant and/or highly similar contigs, they were then clustered at a sequence similarity threshold value of 90% using CD-HIT-EST. The final transcriptome library had 72,474 transcripts with the longest length of 16,072 bp, an average transcripts size of 803 bp, and N50 of 1,377 bp (Table 1). Using Bowtie2, 87.6% of the trimmed reads were mapped back to the transcripts (S2 Table). Based on BUSCO analysis, 89.5% of transcripts were identified as complete (2083 out of 2326 BUSCO genes) (S1 Fig). Those results confirmed that the assembly data were reliable.

**Table 1:** Summary of assembled contigs by Trinity and clustered transcripts with 90% similarity.

Item	Contigs	Transcripts
Number of assembled contigs	76,601	72,474
≤ 500 bp	40,793 (53.3%)	39,521 (54.5%)
501–1000 bp	15,564 (20.3%)	14,625 (20.2%)
1001–2000 bp	12,930 (16.9%)	11,849 (16.3%)
2001–5000 bp	7,002 (9.1%)	6,210 (8.6%)
> 5001 bp	312 (0.4%)	269 (0.4%)
Longest length of contig (bp)	16,072	16,072
Average length of contig (bp)	827	803
N50 (bp)	1,428	1,377
Total length of contigs (bp)	63,383,628	58,180,257

### Functional annotation and classification

**Fig 2:**
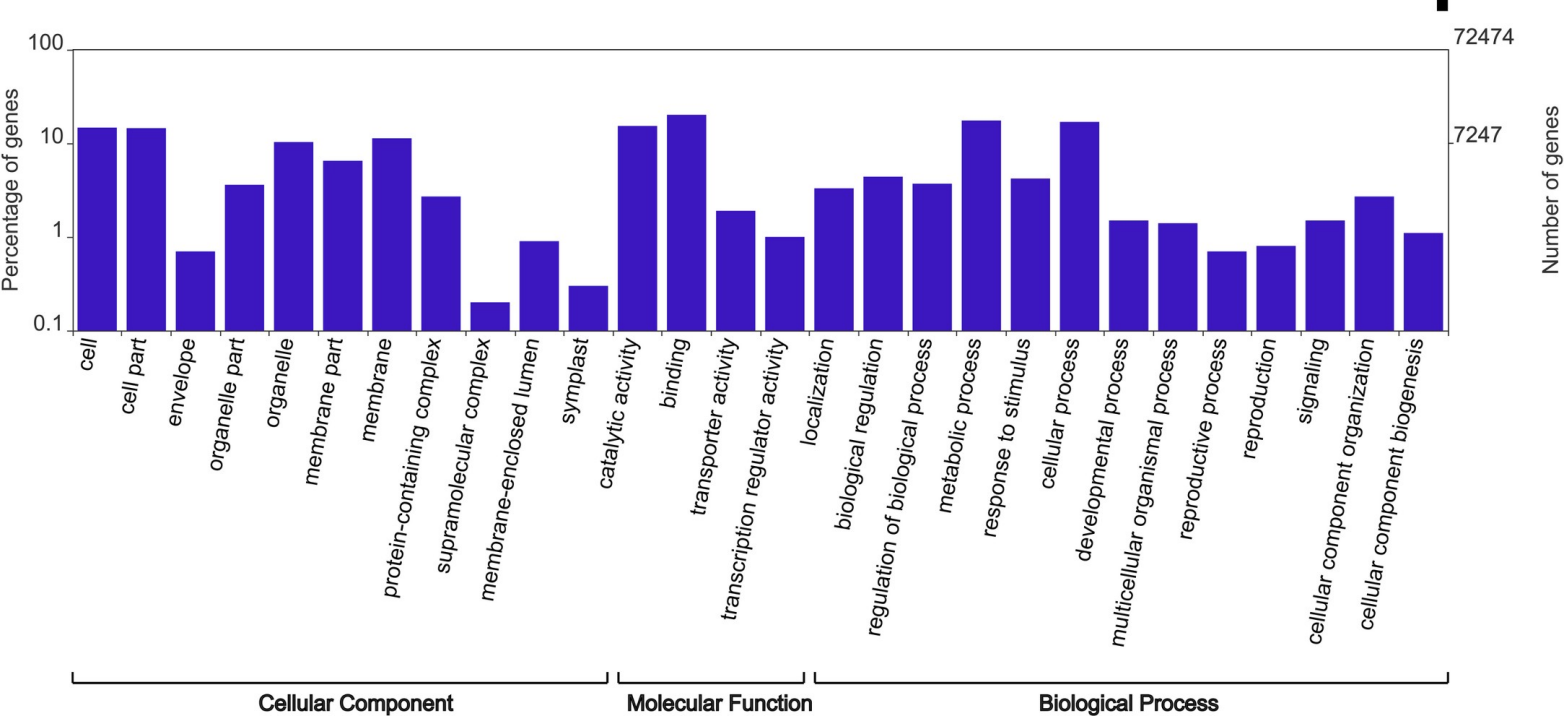
Histogram of Gene Ontology (GO) classification of transcripts into three main categories, including cellular component, molecular function, and biological process. Gene Ontology classification was performed by Web Gene Ontology Annotation Plot.

A total of 33,024 (45.6%) transcripts were annotated against *S*. *lycopersicum* protein database [23] using BLASTx. Gene Ontology analysis was performed to classify transcripts based on the possible functions. A total of 25,256 transcripts were classified into three main categories: cellular component, molecular function, and biological process (Fig 2). Among the three categories, the molecular function category included the highest number of transcripts (20,832), following the biological process category (16,777) and the cellular component category (15,398). In the molecular function category, the top two categories were “binding” (20.1%) and “catalytic activity” (15.3%). In the biological process category, “metabolic process” (17.5%) and “cellular process” (16.9%) were the most abundantly represented. Under the cellular component category, most of the transcripts were involved in “cell” (14.7%), “cell part” (14.5%), and “membrane” (11.3%).

### Differentially expressed genes under water deficit stress

Gene expression level was analyzed by aligning the trimmed paired-reads to the assembled transcripts. Approximately 88% of the trimmed reads were mapped to the transcripts (S2 Table). Based on the principal component analysis (S2 Fig), one library from each treatment on each day was considered as an outlier, therefore twelve libraries were used for further analyses. Differentially expressed genes were identified by comparing gene expression levels between control and stressed plants at each time point.

**Fig 3:**
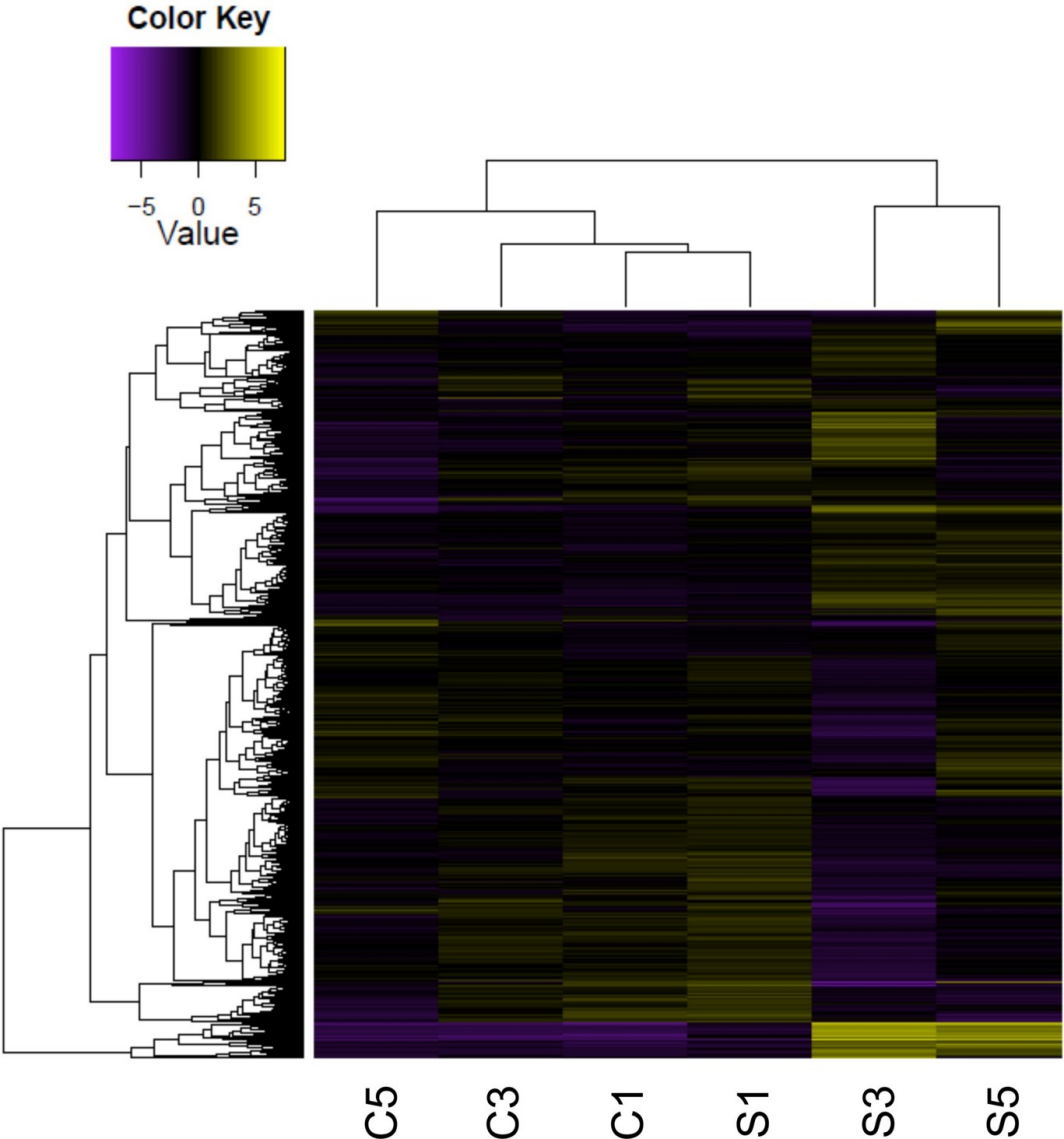
Hierarchical clustering analysis of Differentially Expressed Genes (DEGs). Rows represent individual genes. Genes that increased and decreased in abundance are indicated in yellow and purple, respectively. C and S correspond to control and stressed plants, respectively, and the numbers indicate the number of days of water withholding. DEGs were identified if a two-fold or greater changes in transcript levels between control and stress plants at each time point and a false discovery rate < 0.05.

**Fig 4:**
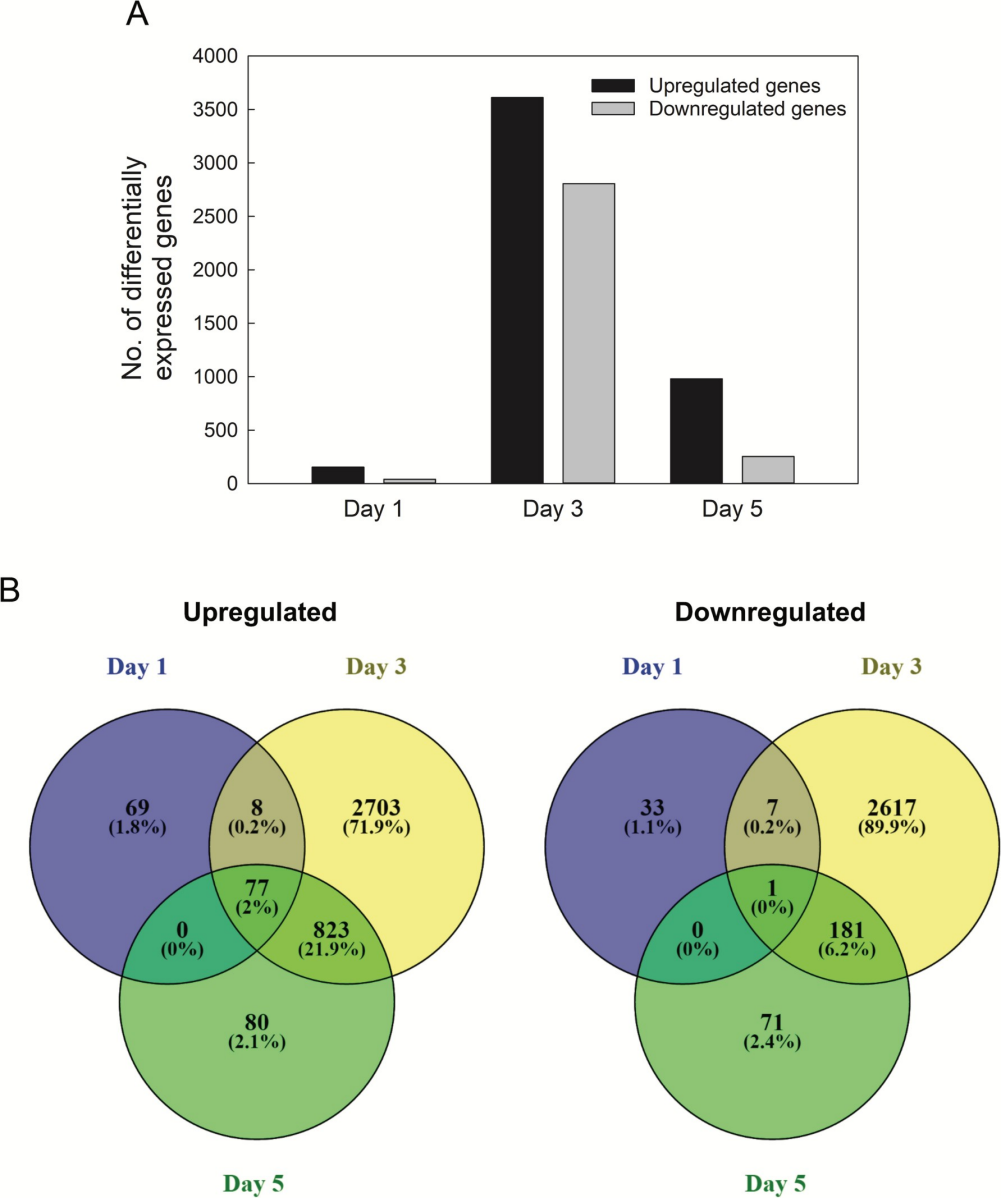
Number of Differentially Expressed Genes (DEGs) under water deficit stress. (A) Number of up or downregulated genes on each day. (B) Venn diagram of upregulated (left) and downregulated (right) genes. DEGs were identified if a two-fold or greater changes in transcript levels between control and stress plants at each time point and a false discovery rate < 0.05.

The results of hierarchical clustering indicated that the difference in gene expression was more prominent at the later stage of the treatment (C3, C5, and S3, S5) than at the early stage (C1 and S1) (Fig 3). This result was also supported by the number of genes that were differentially expressed under water deficit. Significantly higher number of genes were found on day 3 (6417 genes) and 5 (1233 genes) than day 1 (195 genes) (Fig 4A). Under water deficiency, 77 DEGs were commonly upregulated on all three time points, while 69, 2703, and 80 DEGs were upregulated uniquely on day 1, 3, and 5, respectively (Fig 4B). Only 1 DEG was downregulated on all three time points, while 33, 2617, and 71 DEGs were downregulated uniquely on day 1, 3, and 5, respectively (Fig 4B). A total of 6670 DEGs represent approximately 9.2% (6670/72,474) of the total expressed transcripts, and 3742 DEGs were finally annotated.

### Enrichment analysis

Gene Ontology enrichment analysis was performed to classify biological functions of DEGs under water deficit (Tables 2 and 3). There were 8, 240, and 15 enriched biological terms from the upregulated process terms in DEGs during water deficit stress (S1 File). One biological process term, sulfur compound metabolic process (GO:0006790), was enriched across all three time points, and 6 and 12 biological process terms were enriched in both day 1 and 3, and day 3 and 5, respectively (Table 2). The common biological process terms in petunia under water deficit are classified into three categories: metabolic process, biological regulation, and transport (Table 2). Metabolic process included sulfate reduction (GO:0019419), sulfate assimilation (GO:0019379), and glutathione catabolic process (GO:0006751). The second category, biological regulation, was associated with homeostatic process (GO:0042592), cell redox homeostasis (GO:0045454), and cellular homeostasis (GO:0019725). The transport category consisted of sulfate transport (GO:0008272) and anion transport (GO:0006820). Enrichment analysis of downregulated genes showed 194 and 88 biological terms on day 3 and 5, while no biological term was enriched on day 1 (S1 File). Sulfur and glutathione metabolic processes (GO:006790 and GO:006749, respectively), and cellular homeostasis (GO:0019725) were enriched among downregulated genes in both day 3 and 5 (Table 3). The enriched molecular function and cellular component terms are shown in S1 File.

**Table 2:** Biological process terms enriched commonly at least two days by upregulated genes under water deficit stress.

Day	Category	GO ID	GO term
Day 1 and 3	Metabolic process	GO:0019419	sulfate reduction
		GO:0019379	sulfate assimilation, phosphoadenylyl sulfate reduction by thioredoxin
		GO:0055114	oxidation-reduction process
	Biological regulation	GO:0042592	homeostatic process
		GO:0045454	cell redox homeostasis
		GO:0019725	cellular homeostasis
Day 3 and 5	Metabolic process	GO:0044272	sulfur compound biosynthetic process
		GO:0006751	glutathione catabolic process
		GO:0042219	cellular modified amino acid catabolic process
	Transport	GO:0098656	anion transmembrane transport
		GO:1902358	sulfate transmembrane transport
		GO:0008272	sulfate transport
		GO:0006820	anion transport
		GO:0015698	inorganic anion transport
		GO:0072348	sulfur compound transport
		GO:0015103	inorganic anion transmembrane transport
		GO:0000101	sulfur amino acid transport
		GO:0015811	L-cystine transport
Day 1, 3, and 5	Metabolic process	GO:0006790	sulfur compound metabolic process

**Table 3:** Biological process terms enriched commonly on day 3 and 5 by downregulated genes under water deficit stress.

Day	Category	GO ID	GO term
Day 3 and 5	Metabolic process	GO:0005975	Carbohydrate metabolic process
		GO:0006749	Glutathione metabolic process
		GO:0042744	Hydrogen peroxide catabolic process
		GO:0006790	Sulfur compound metabolic process
		GO:0046942	Carboxylic acid transport
	Biological regulation	GO:0019725	Cellular homeostasis

### Antioxidant activities and sulfur metabolism under water deficit

**Fig 5:**
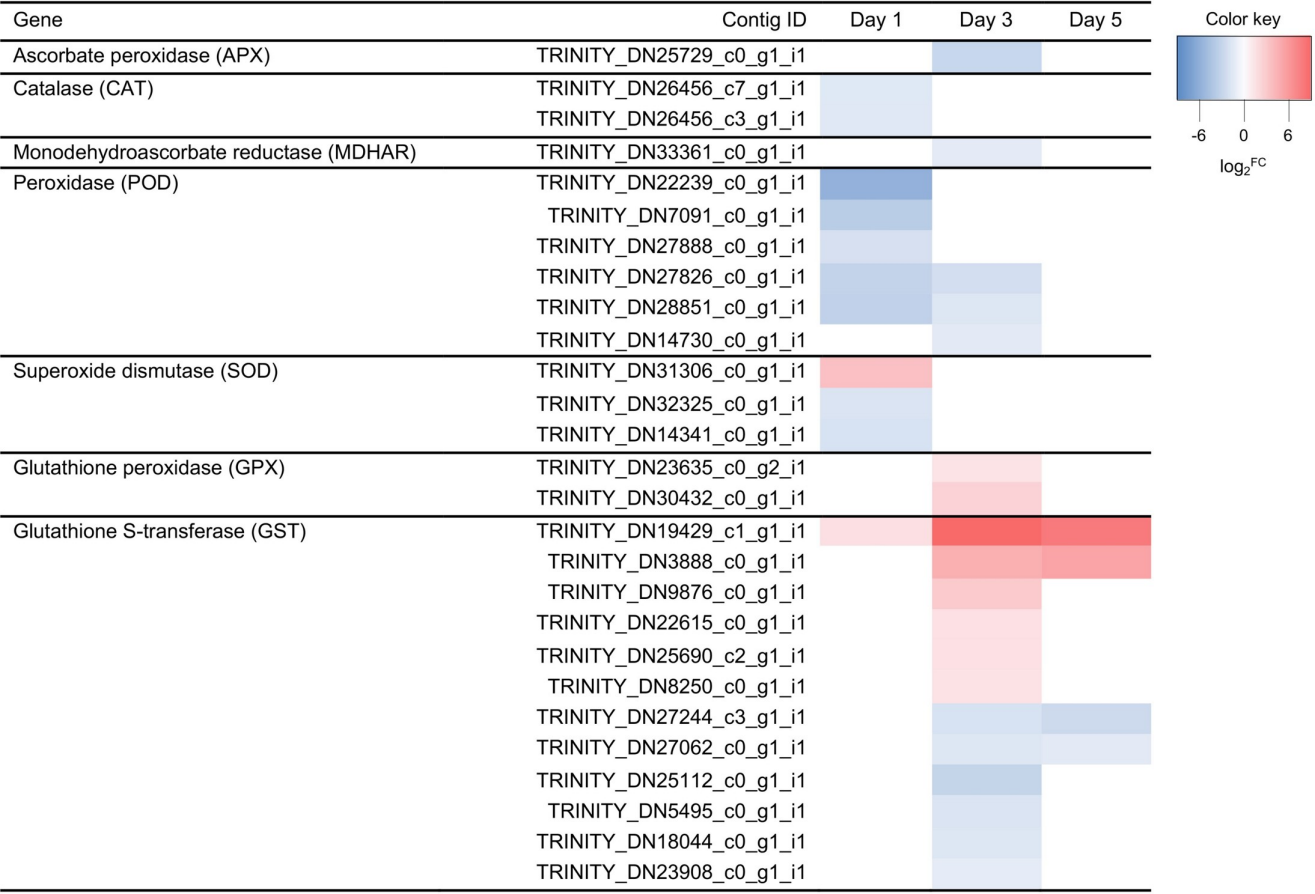
Heatmap of differentially expressed genes encoding antioxidant enzymes under water deficit stress. Expression values of genes are presented as Trimmed Mean of M value (TMM)-normalized log_2_^fold change (stressed plant/control)^. Pink and blue colors indicate up and downregulated transcripts under water deficit stress, respectively.

GO enrichment analysis showed that homeostasis processes including ‘cell redox homeostasis’ and sulfur/sulfate-related processes were dominant under water deficit stress (Tables 2 and 3). Genes encoding antioxidant enzymes were differentially expressed under water deficit, including ascorbate peroxidase (APX), catalase (CAT), monodehydroascorbate reductase (MDHAR), peroxidase (POD), superoxide dismutase (SOD), glutathione peroxidase (GPX), and glutathione-S transferase (GST) (Fig 5). Most of APX, CATs, MDHAR, PODs, and SODs were downregulated on day 1 and/or 3, but not differentially expressed on day 5 compared to control (Fig 5). Glutathione (GSH)-linked enzymes, GPXs and GSTs, were also identified in petunia. All of GPXs were upregulated only on day 3, while GSTs had either upregulated or downregulated expression patterns under water deficit (Fig 5). Half of GSTs were upregulated, and the other half were downregulated. One GST (TRINITY_DN19429_c1_g1_i1) was the most dominant at all three time points, and its expression level was 290 and 139-fold higher than that of control on day 3 and 5, respectively (Fig 5). In contrast, downregulated GSTs had at least -6-fold changes compared to control (Fig 5).

**Fig 6:**
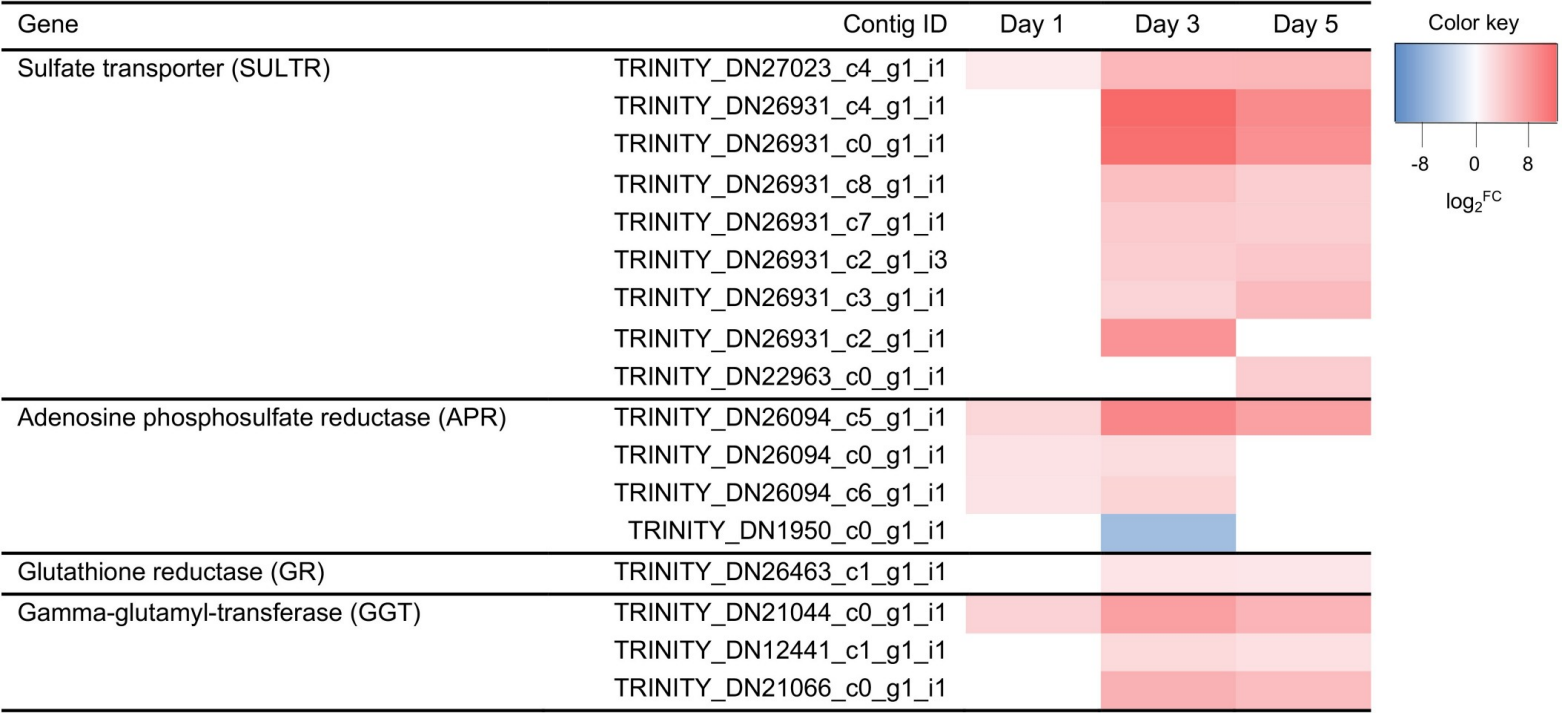
Heatmap of differentially expressed genes involved in sulfur metabolisms under water deficit stress. Expression values of genes are presented as Trimmed Mean of M value (TMM)-normalized log_2_^fold change (stressed plant/control)^. Pink and blue colors indicate up and downregulated transcripts under water deficit stress, respectively.

Fig 6 shows genes involved in sulfur metabolic processes under water deficit in petunia. Nine sulfate transporters (SULTRs) were induced under water deficit, and one of them (TRINITY_DN27023_c4_g1_i1) were upregulated at all three days (Fig 6). Adenosine phosphosulfate reductase (APR) and glutathione reductase (GR) are an enzyme in sulfur assimilation and glutathione recycling pathways, respectively. Three APRs and one GR were upregulated under water stress (Fig 6). Gamma-glutamyl-transferase (GGTs), an enzyme of extra-cytosolic glutathione degradation, were also upregulated under water deficit (Fig 6).

### Hormone signal transduction in response to water deficit stress

**Fig 7:**
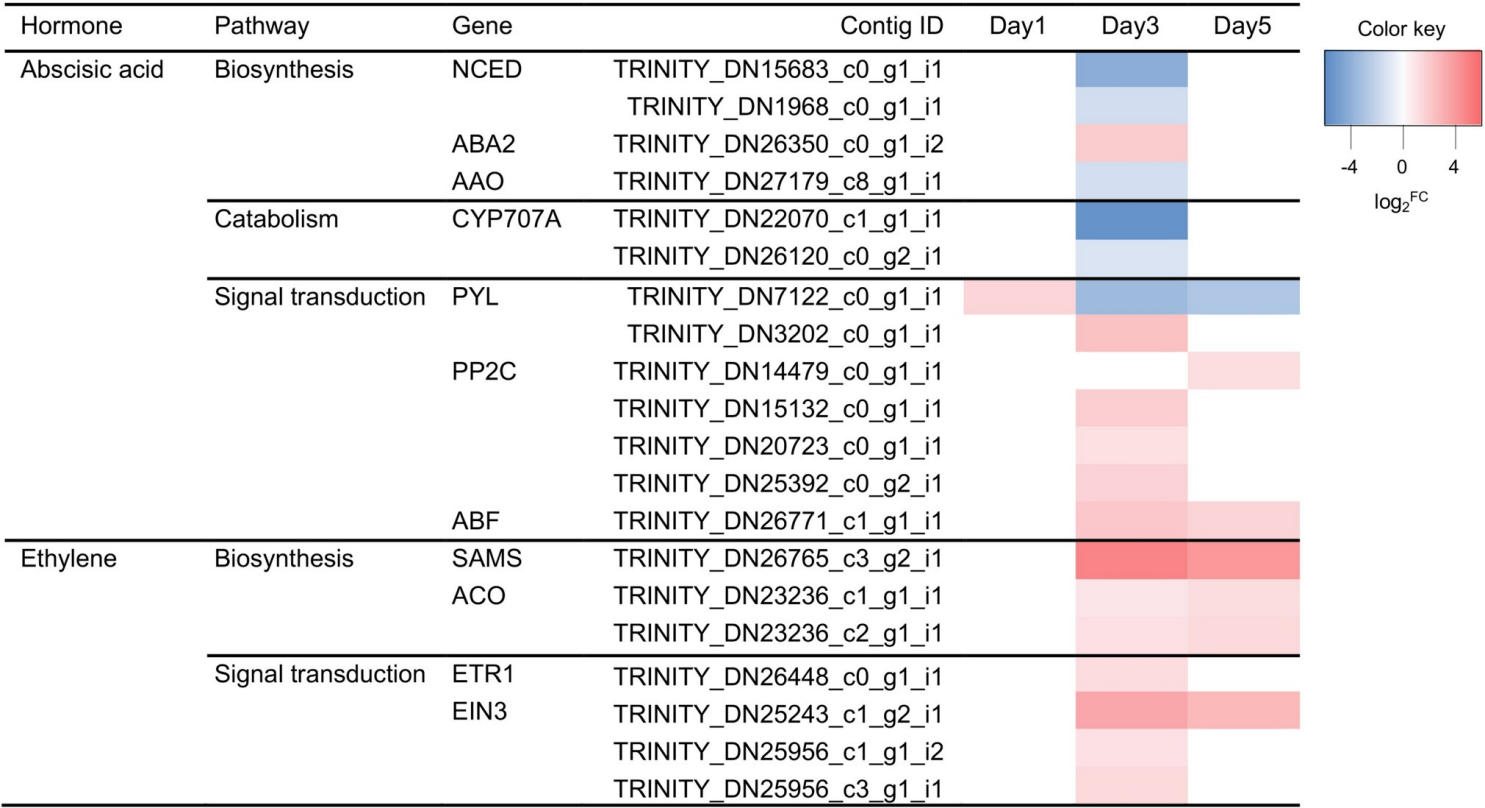
Heatmap of differentially expressed genes involved in abscisic acid and ethylene biosynthesis and signal transduction pathways under water deficit stress. Expression values of genes are presented as Trimmed Mean of M value (TMM)-normalized log_2_^fold change (stressed plant/control)^. Pink and blue colors indicate up and downregulated transcripts under water deficit stress, respectively.

**Fig 8:**
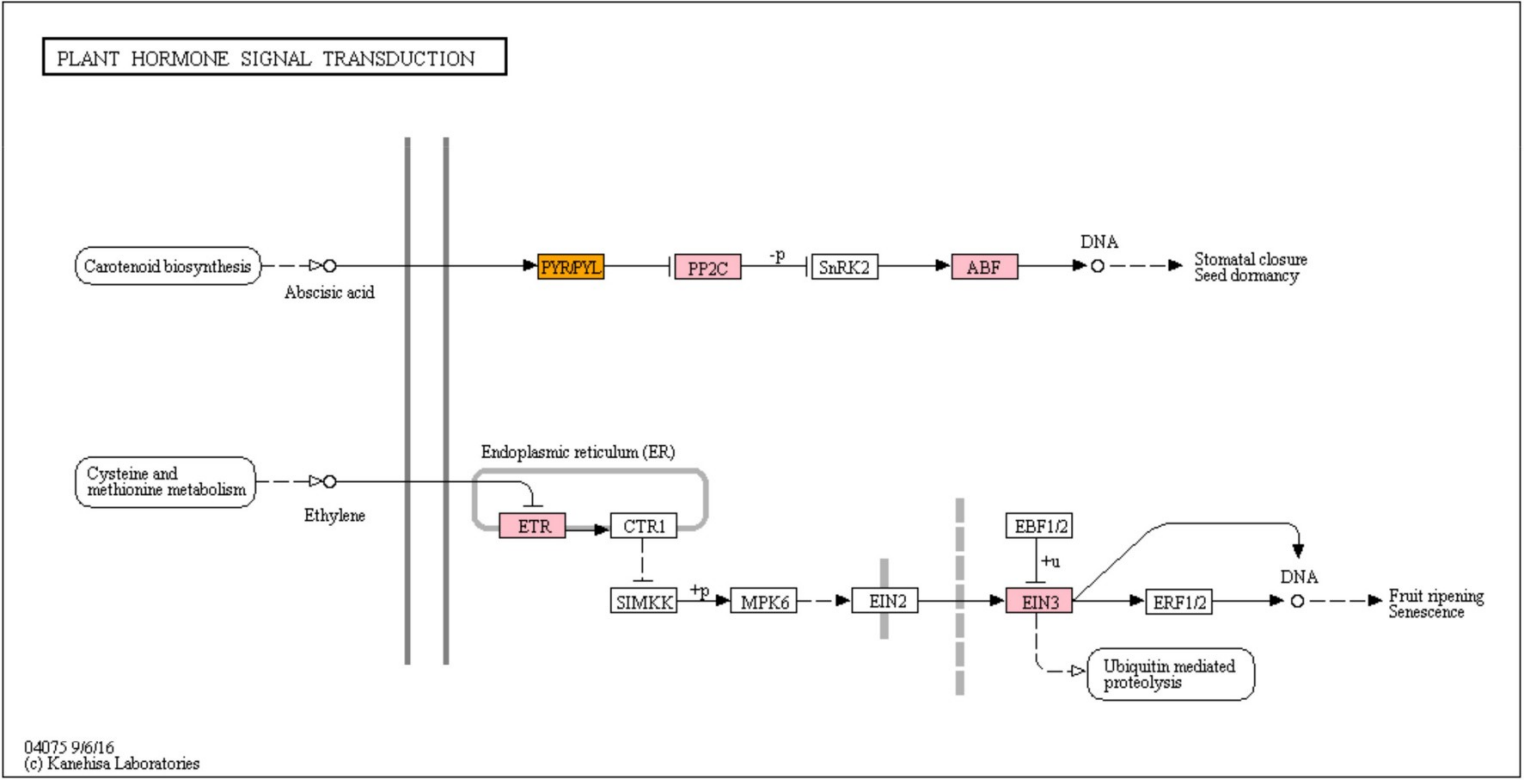
Abscisic acid and ethylene signal transduction Kyoto Encyclopedia of Genes and Genomes pathway. Pink boxes represent genes that were upregulated under water deficit stress, while the orange box indicate genes which were up and downregulated during five days of water deficit.

Under water deficit, key genes of abscisic acid (ABA) biosynthesis [9-*cis*-epoxycarotenoid dioxygenase (NCED), xanthoxin dehydrogenase (ABA2), and ABA aldehyde oxidase (AAO)] and catabolism [ABA 8’-hydroxylase (CYP707A)] were differentially expressed on day 3 (Fig 7). Only one of them (ABA2) were upregulated, while others were downregulated three days after withholding water. In addition, the expression of seven genes involved in ABA signal transduction [two pyrabactin-like (PYLs), four phosphatase 2C (PP2Cs), and one ABA response element binding factor (ABF)] were upregulated except one of PYLs (Figs 7 and 8). This gene was induced on day 1, but its expression was downregulated on day 3 and 5. It was also identified DEGs involved in ethylene biosynthesis and signal transduction (Figs 7 and 8). Three ethylene synthesis-related genes [S-adenosylmethionine synthetase (SAMS) and 1-aminocyclopropane-1-carboxylic acid oxidase (ACO)] were induced on days 3 and 5. Also four signal transduction-related genes [ethylene response 1 (ETR) and three ethylene insensitive 3 (EIN3)] were upregulated; three of those genes were upregulated on day 3 and one of EIN3 was upregulated on days 3 and 5 (Figs 7 and 8).

### Transcription factors responding to water deficit stress

**Fig 9:**
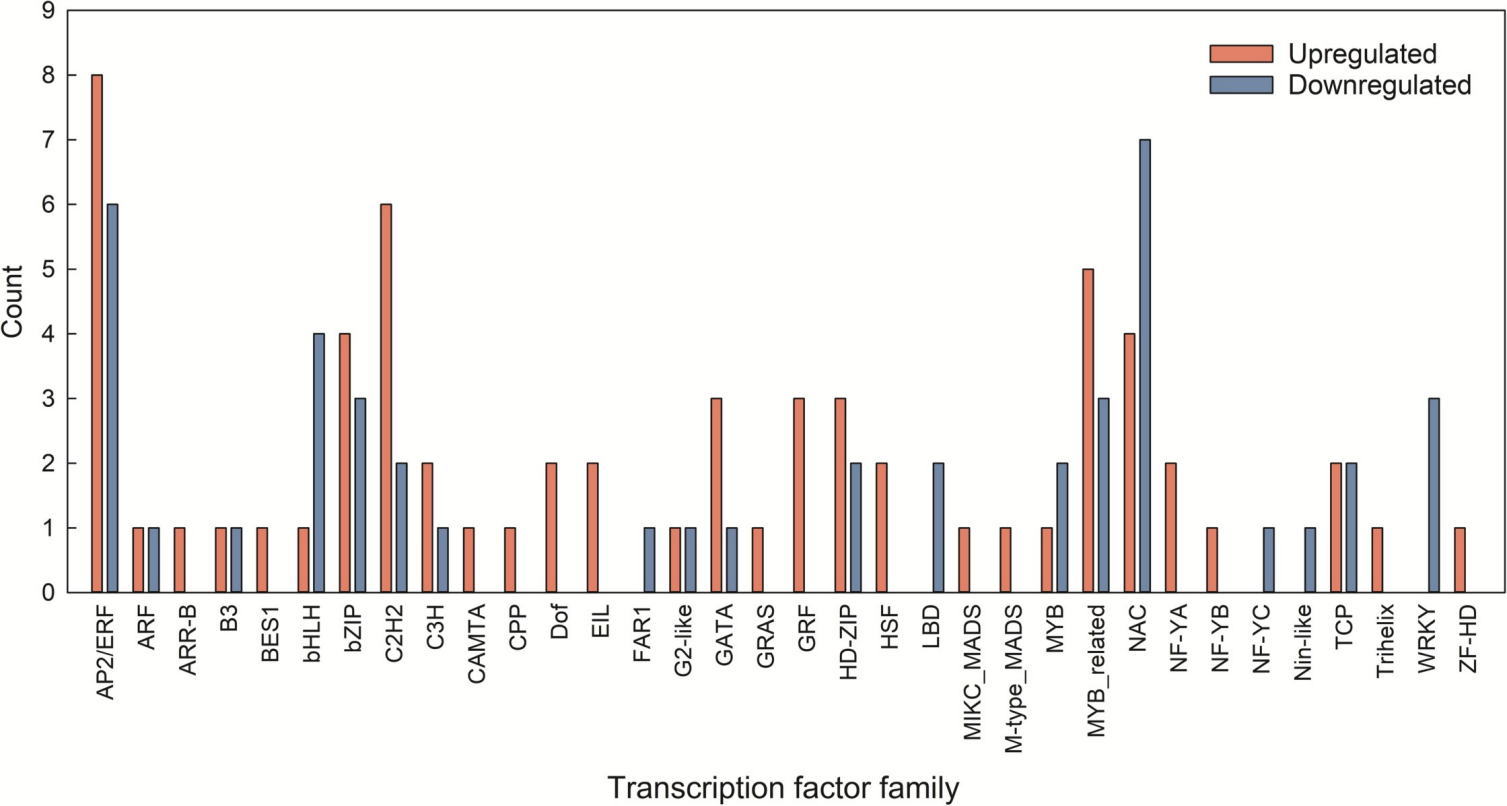
Distribution of differentially expressed Transcription Factor (TF) families. Pink bars indicate upregulated TFs and blue bars indicate downregulated TFs under water deficit stress.

In response to dehydration, 63 genes encoding TFs were upregulated and 44 were downregulated (Fig 9). These TFs were classified into 34 families based on their putative DNA-binding domains. The most abundant TF families were APETALA 2/ethylene response factor (AP2/ERF)(14), no apical meristem, Arabidopsis *thaliana* activating factor, and cup-shaped cotyledon (NAC)(11), myeloblastosis-related (MYB-related)(8), Cys_2_-His_2_-like fold group (C2H2)(8), basic leucine zipper (bZIP)(7), followed by basic helix-loop-helix (bHLH)(5), homeodomain leucine zipper (HD-ZIP)(5), teosinte branched, cycloidea, and PCF (TCP)(4), and GATA-binding factor (GATA)(4). The most abundant upregulated TF families were AP2/ERF (8), C2H2 (6), and MYB-related (5), and the most abundant downregulated TF families were NAC (7), AP2/ERF (6), and bHLH (4). Among a total of 63 upregulated TFs, only one TF, growth-regulating factor (GRF) was upregulated on day 1, and other TFs were upregulated on day 3 and/or 5 (S3 Fig). All downregulated TFs were found on day 3 and/or 5, while no TFs were downregulated on day 1 (S3 Fig). In addition, 101 out of 107 differentially expressed TFs were found on day 3 (S3 Fig).

### Validation of differentially expressed genes using qPCR

**Fig 10:**
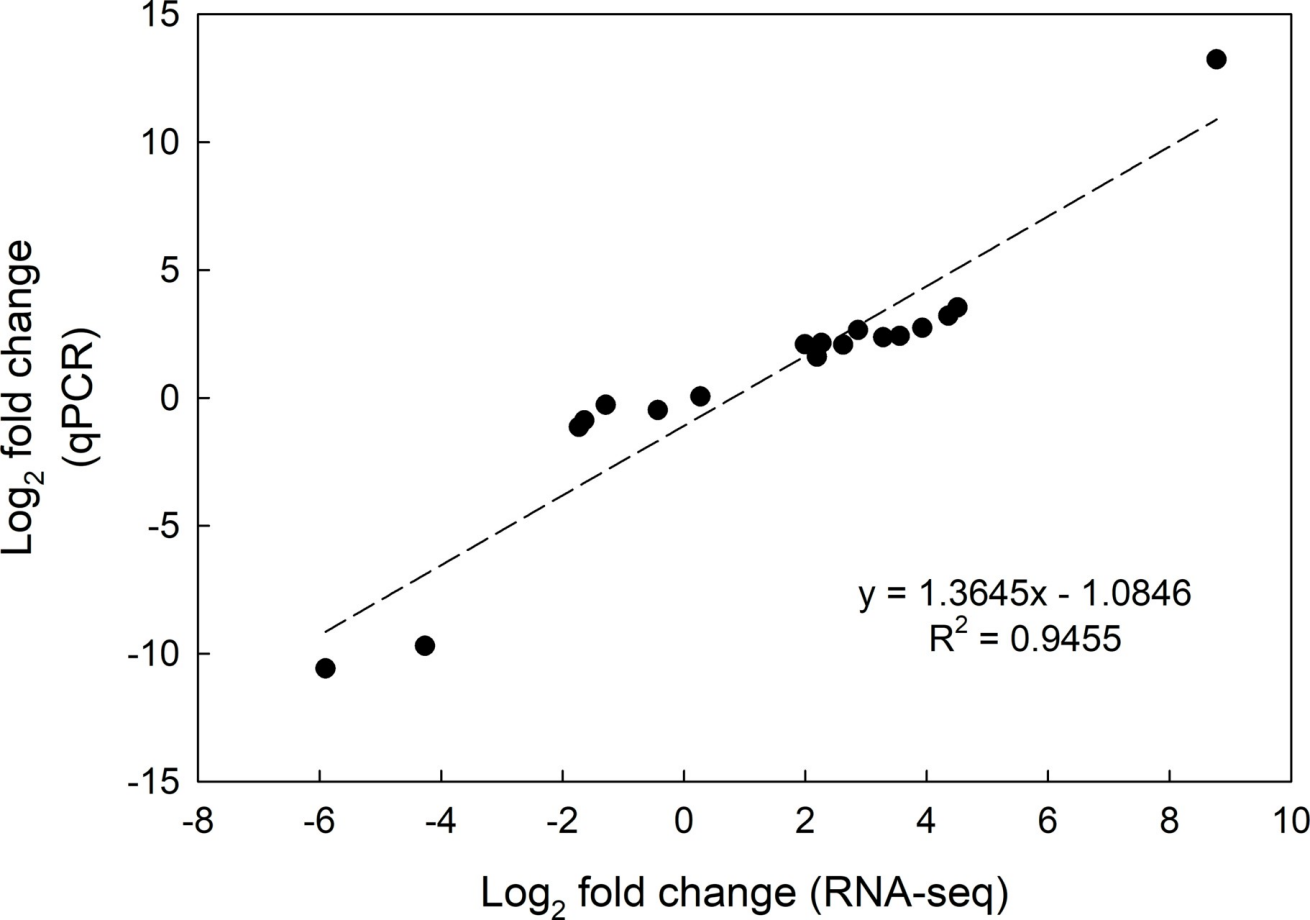
Correlation analysis of the gene expression fold change between RNA-seq and qPCR.

To confirm RNA-seq data, qPCR was performed with five target genes which showed distinct expression patterns over the three time points. The fold changes of gene expression in the target genes had a similar trend with those from RNA-seq analysis (R^2^ = 0.9455, Fig 10), which indicated a high reliability of RNA-seq data.

## Discussion

Plants have evolved to overcome water deficit stress, such as changes in physiological and biochemical status. These changes are associated with the differential expression of functional genes. RNA-seq was performed using leaf tissue of *P*. ×*hybrida* to analyze transcriptomic changes under water stress. Five days after withholding water, no visual wilting was observed, but stressed plants reduced stomatal conductance by 38% compared to control (Fig 1). This physiological change of stomatal closure indicated that plants perceived and responded to water stress within five days. Leaves under water deficit stress for 1, 3, and 5 days were used for RNA-seq, and each sampling time point could be defined as immediate, early, and late stages of water deficit, respectively. These time-course transcriptomic data demonstrated the global gene expression profile under water deficit stress in *P*. ×*hybrida*.

In total, 262,427,357 high-quality sequencing reads were assembled into 76,601 contigs, and a total of 72,474 transcripts were generated by the program CD-HIT-EST (Tables 1 and S2). The average length of 803 bp and N50 of 1,377 bp are comparable to the results from other *de novo* transcriptome assemblies [24–29]. BLASTx results were obtained for 45.6% transcripts against *S*. *lycopersicum* protein database [23]. The petunia transcript data has a similar distribution of GO compared to *de novo* assembled sequences from other species subjected to water deficit [24, 30]. This study provided more than 33,000 annotated transcripts, which could be utilized for further research in petunia. Additionally, the qPCR results showed that RNA-seq data was reliable (Fig 10).

Of the three time points, the fewest DEGs were found on day 1 (195) and the most DEGs were found on day 3 (6417) (Fig 4A). The same pattern was observed in the number of TFs (S3 Fig). The hierarchical clustering of samples also showed that the expression profile of stressed plants on day 1 was closer to those of control samples rather than other stressed samples on day 3 and 5 (Fig 3). These results suggested that one day of withholding water might have initiated the water deficit stress response, and gene expression was more active three days after water was withhold. Results of the reduced number of DEGs on day 5 indicated that plants might have acclimated to water deficit. The physiological changes of stomatal closure, the numbers of DEGs and TFs on each time point, and the hierarchical clustering pattern coincided with the number of differentially expressed genes on day 1, 3, and 5 as the immediate, early, and late stages of water deficit. The more DEGs were found at least three days after water withholding, showing more genes tended to be consistently upregulated or downregulated to share mechanisms between the early and late stages against water deficit stress. Most of the other transcriptomic analyses under abiotic stress had more upregulated DEGs than downregulated [25, 28, 31–35].

As water deficit stress occurs, the level of reactive oxygen species (ROS) is increased due to the disturbance between generation and quenching of ROS under stress conditions. Reactive oxygen species causes peroxidation of membrane lipid, degradation of proteins and nucleic acid, cellular oxidative damages, and disruption of cellular homeostasis. To mitigate this harmful effect, plants initiate the antioxidant activity to scavenge ROS [36]. Plant cells trigger a complex antioxidant system with a variety of enzymes and non-enzymatic compounds to mitigate the detrimental effects of ROS. Seven antioxidant enzymes (APX, CAT, MDHAR, POD, SOD, GPX, and GST) were identified in this study, indicating that antioxidant systems are involved in the early response of water deficit in petunia (Fig 5). Most of the genes were downregulated at the immediate stage, however the expression of GSH-linked enzymes were increased at the early stage of water deficit (Fig 5). As water stress increased, there were more upregulated GSTs than downregulated GSTs (Fig 5). Tomato has shown to have 90 GST genes, and most of them were highly induced in response to various biotic and abiotic stresses [37]. It is predicted that tomato GSTs interact with other glutathione-dependent enzymes, such as GPX, GR, GGT, and glutathione synthetase [37].

Sulfur is a key component for the synthesis of glutathione, which plays in the cellular redox balance and mitigates damages by ROS [38], and sulfur-containing metabolites pathways were upregulated under water deficit in Chinese cabbage leaves [39]. Wu *et al*. [40] suggested that sulfur plays a critical role in the early response to water deficit in *Alhagi sparsifolia* primary roots. In this study, GO enrichment analysis showed that sulfur/sulfate-related metabolic processes represented among DEGs in water deficit responses (Tables 2 and 3). Sulfur is mostly taken up by plants in a sulfate form, and the contribution of sulfate transporters (SULTRs) in abiotic stress tolerance has been investigated [41]. In *Medicago truncatula*, *MtSULTR3* were induced in root and shoot at mild, moderate, and severe drought conditions [42]. Gallardo *et al*. [41] reviewed that *AtSULTR4* genes were significantly enhanced in leaves by water deficit and salinity in Arabidopsis. Because SULTR4s were involved in the efflux of sulfate stored in the vacuoles, they are considered to play a role in sulfur metabolism when sulfate uptake is limited due to adverse environments [43]. In our petunia data, homologs of SULTR3 and 4 (TRINITY_DN22963_c0_g1_i1 and TRINITY_DN27023_c4_g1_i1, respectively) were upregulated under water deficit, especially SULTR4 at all three time points, and more SULTRs were upregulated in petunia as water deficit prolonged (Fig 6). Our study indicates that several upregulated SULTRs may contribute to adjust sulfate distribution when subjected to water deficit stress in petunia.

Sulfate is converted to adenosine phosphosulfate (APS), and APS is catalyzed by APS reductase (APR) entering the primary sulfur assimilation pathway or phosphorylated by APS kinase into the secondary assimilation pathways [38]. In the primary assimilation pathway, GSH is formed as reduced GSH from cysteine, and GSH functions as a non-enzymatic antioxidant within the cell, resulting in oxidized glutathione (GSSG). Then, GSSG is recycled back to GSH by glutathione reductase (GR) [38, 44]. Glutathione reductase is responsible for the recycling of GSSG and maintaining the supply of GSH [44]. Transgenic plants with high activity of GR showed increases in antioxidant capacity, which rendered tolerance to abiotic and/or biotic stresses in tobacco [45], poplar [46], and cotton [47]. GGTs are the only enzymes to hydrolyze the gamma-glutamyl bond in GSH, and they are known to be involved in GSH recycling in extra-cytosolic (apoplastic and vacuolar) [48]. The expression level of GGTs were increased in *Phormium tenax* under water deficit stress [32], and the inhibition of GGT resulted in enhanced ROS generation in Cd-treated seedling [49]. Trentin *et al*. [50] showed that *ggt1* knockout mutant had higher ROS production compared to wild type Arabidopsis. It is hypothesized that GSH recycling by GGTs plays as a signal to transfer redox information between symplast and apoplast, however the significance of this recycling is not fully understood yet [48]. In this study, APRs, GR, and GGTs had increased expression levels on almost all time points (Fig 6). These results suggest that sulfate enters into the primary sulfur assimilation by APRs to produce GSH for homeostasis processes and its level is maintained by GR and GGTs through GSH recycling systems under water deficit response in petunia.

Phytohormones play essential roles in plant acclimation and adaptation to cope with adverse environmental changes [51]. In this study, the expression levels of genes involved in hormone biosynthesis and signal transduction were changed, including ABA, ethylene, jasmonic acid, and salicylic acid. Abscisic acid is known as a major hormone playing crucial roles in abiotic stress responses by inducing stomatal closure and regulating gene expression [52]. Previous studies have shown that many genes involved in ABA biosynthesis and signaling were upregulated under water deficit [28, 29, 53, 54]. A rate-limiting enzyme in ABA biosynthesis was induced and its expression level was gradually increased as dehydration prolonged in pear [4]. However, in this research, the expression of two NCEDs were not changed or downregulated under water deficit (Fig 7). Instead, ABA 8’ hydroxylase (CYP707A), a catabolic enzyme of ABA, was downregulated under water deficit (Fig 7). Takeuchi *et al*. [55] suggested that inhibition of CYP707A may control ABA mechanisms and increase water deficit tolerance, as shown in Arabidopsis and barley [56, 57]. Abscisic acid binds to the PYR/PYL/RCAR receptors in order to inhibit the activities of PP2Cs [52]. Tomatoes grafted onto drought tolerant rootstocks enhanced tolerance to water deficit by upregulating genes in ABA signal transduction (PYR/PYL and PP2C), not genes in ABA biosynthesis [58]. In this study, a PYL gene was upregulated by approximately 3-fold after one day of water deficit, implying that ABA signal transduction was triggered at immediate stage of water deficit in petunia. In addition, the expression of the key components in the ABA signal transduction pathway, PP2C and ABF, were upregulated by 2.3 to 4.4-fold after 3 and 5 days of water deficit treatment. This result suggested that the ABA signal transduction was initiated at immediate stage of water deficit, and ABA regulated stress responses such as stomatal closure on the fifth day after withholding water.

Ethylene is involved in plant growth and development, such as senescence and fruit ripening [59]. In this study, two ethylene biosynthetic enzymes (SAMS and ACO) were upregulated day 3 and 5. Genes involved in ethylene signaling (ETR1 and EIN3) were also upregulated on day 3 and/or 5, activating diverse ethylene responses during the early and late stages of water deficit. Especially, EIN3 induces expression of ethylene responsive factor 1 (ERF1), which is a major TF in plant defense responses in the ethylene signaling pathway [60]. Egea *et al*. [61] noted that the expression level of ACOs and ERFs were higher in drought-tolerant wild tomato than cultivated tomato, and those genes were also upregulated under water deficit in drought-tolerant tomato. Overexpression of *S*. *lycopersicum SlSAMS1* conferred water deficit and salt tolerance by increasing antioxidant activity, photosynthetic capacity, and water-retention capacity [62]. Taken together, the alternation of ethylene biosynthesis and signaling genes seems to be related to the early response of water deficit including stomatal closure in petunia.

Several TFs have crucial roles in regulating downstream gene expression, and many TFs have been identified in transcriptome analysis under abiotic stresses [28, 29, 63]. The members of AP2/ERF, NAC, MYB-related, C2H2, and bZIP TF families were differentially expressed as found in other studies [7, 28, 64]. Especially, AP2/ERF family was mostly differentially expressed in petunia; there were eight upregulated and six downregulated genes in this study (Fig 9). Previous studies have shown that manipulation of AP2/ERF TFs conferred enhanced abiotic stress tolerance. For example, overexpression of an AP2/ERF from *Chrysanthemum morifolium*, *CmERF053*, enhanced water deficit stress tolerance in Arabidopsis by showing significantly lower rate of water-loss [65], and overexpression of a *Oryza sativa* AP2/ERF TF (*OsEREBP1*) enhanced survival under water deficit and submergence in rice [66]. *JcERF2* from *Jatropha curcas* L. enhanced water deficit and salt tolerance in transgenic tobacco with increased free proline and soluble carbohydrate accumulation [67].

Notably, one member of growth regulating factors (GRFs, TRINITY_DN25774_c0_g2_i1) was upregulated on day 1, while all other TFs were up or downregulated on day 3 and 5 of water deficit (S2 Fig). Growth regulating factors are plant-specific TFs that were originally identified in stem and leaf development, but recently they are suggested as candidate factors for the coordination of plant growth under adverse environmental conditions [68, 69]. Khatun *et al*. [69] reported that differential expression of GRFs in tomato was observed under salt, drought, heat, cold, ABA, and jasmonic acid treatments, predicting possible functions of GRFs in stress responses. A previous study in the overexpression of *AtGRF1* and *AtGRF3* suggested that they contributed to regulating various biological processes involved in defense response and disease resistance in Arabidopsis [70]. In this research, it is considered that GRFs play roles in the immediate response of water deficit, and they could be good candidates for elucidating water deficit response networks in petunia.

## Conclusion

With high-throughput sequencing and *de novo* assembly, the comprehensive transcriptome data was obtained from *P*. ×*hybrida* ‘Mitchell Diploid’ grown under water deficit stress. Analysis of DEGs provided an excellent database for future genetic and genomic analyses as well as insights into the understanding potential mechanisms of water deficit stress response in petunia. Genes associated with tolerance to water deficit stress were identified and their expression profiles were analyzed over three time points of water deficit. The DEGs included well-known TFs such as AP2/ERF, NAC, MYB-related, and C2H2, and AP2/ERF family were the most abundant under water deficit in petunia. In addition, metabolic processes, sulfur metabolic pathway for antioxidant activity and hormone signal transduction of ABA and ethylene, were enriched in *P*. ×*hybrida*. Changes in hormone signal transduction may function upstream of water deficit response and then lead to gene expression and physiological changes, such as stomatal closure. These findings will provide a better understanding on molecular mechanisms underlying water deficit tolerance in *P*. ×*hybrida*, ultimately leading to identify the genes regulating response to water deficit stress. Furthermore, the knowledge obtained from this research can be applied to develop staple crops with enhanced tolerant traits to water deficit stress.

## Supporting information

S1 Table Primers used for qPCR analyses. (DOCX)

S2 Table The number of sequenced, trimmed, and mapped reads (50 bp-paired end). (DOCX)

S1 Fig The completeness of *de novo* assembled transcripts using Benchmarking Universal Single-Copy Orthologs (BUSCO). (TIF)

S2 Fig Principal component analysis plot of the RNA-seq libraries. C and S correspond to control and stressed plants, respectively, and the numbers indicate the number of days after withholding water. Gray-circled samples are considered as outliers, and they were excluded for further analyses.
(TIF)

S1 File Biological terms enriched under water deficit stress. (ZIP)

S3 Fig Heatmap of differentially expressed TFs under water deficit. Upregulated (left and pink) and downregulated (right and blue) TFs.
(TIF)
