# Supplementary material for: Time-course transcriptomic analysis of Petunia ×hybrida leaves under water deficit stress using RNA sequencing
Source: PLoS One. 2021 Apr 26;16(4):e0250284. doi: 10.1371/journal.pone.0250284 (PMC8075263; doi:10.1371/journal.pone.0250284)
Supplement: S1 Table — (DOCX) [file pone.0250284.s001.docx]

S1 Table. Primers used for qPCR analyses.

| Gene ID | Primer sequence | Product length (bp) |
| --- | --- | --- |
| Internal control (*PhEF1α*) | F: CCTGGTCAAATTGGAAACGG  R: CAGATCGCCTGTCAATCTTGG | 103 |
| TRINITY_DN28967_c0_g1_i1 | F: AGTGACAGGACCTTGGTAGA  R: CCACAGGGATTTCTCCGATATT | 107 |
| TRINITY_DN30261_c0_g1_i1 | F: GAAGCTGAAGGAGACCACTAAC  R: TACTCCATGAGAAAGACAGTGAATAC | 103 |
| TRINITY_DN27512_c3_g1_i2 | F: TGACCGTTATCAATACCCAACTT  R: CCACTAATAGCACTCCCACAAA | 104 |
| TRINITY_DN25243_c1_g2_i1 | F: GGTCCTGTTAGCTTGCTACATT  R: CATAAGGAGCCCAAAGAGAATCA | 103 |
| TRINITY_DN26771_c1_g1_i1 | F: AATCTGAGCCGTTGGATTCAG  R: GTGGTTTACTGGGACCACTTT | 110 |
