# Supplementary material for: Time-course transcriptomic analysis of Petunia ×hybrida leaves under water deficit stress using RNA sequencing
Source: PLoS One. 2021 Apr 26;16(4):e0250284. doi: 10.1371/journal.pone.0250284 (PMC8075263; doi:10.1371/journal.pone.0250284)
Supplement: S2 Table — (DOCX) [file pone.0250284.s002.docx]

S2 Table. The number of sequenced, trimmed, and mapped reads (50 bp-paired end).

| Day | Treatment | Rep. | No. of reads | No. of reads after trimmed | No. of reads mapped |
| --- | --- | --- | --- | --- | --- |
| Day 1 | Control | 1 | 11,705,939 | 11,669,651 | 10,089,705 (86.5%) |
|  |  | 2 | 12,639,684 | 12,592,917 | 11,014,104 (87.5%) |
|  |  | 3 | 16,604,788 | 16,543,350 | 14,679,300 (88.7%) |
|  | Stressed | 1 | 15,116,700 | 15,071,350 | 13,311,211 (88.3%) |
|  |  | 2 | 16,083,109 | 16,023,601 | 14,214,999 (88.7%) |
|  |  | 3 | 17,698,038 | 17,639,634 | 15,333,796 (86.9%) |
| Day 3 | Control | 1 | 18,128,347 | 18,061,272 | 15,529,575 (86.0%) |
|  |  | 2 | 12,954,123 | 12,910,079 | 10,959,056 (84.9%) |
|  |  | 3 | 14,756,225 | 14,683,919 | 12,845,284 (87.5%) |
|  | Stressed | 1 | 15,778,128 | 15,707,126 | 13,827,321 (88.0%) |
|  |  | 2 | 11,907,904 | 11,857,891 | 10,196,479 (86.0%) |
|  |  | 3 | 14,329,417 | 14,274,965 | 12,316,744 (86.3%) |
| Day 5 | Control | 1 | 18,716,488 | 18,662,210 | 16,918,912 (90.7%) |
|  |  | 2 | 8,898,370 | 8,874,344 | 7,747,028 (87.3%) |
|  |  | 3 | 14,483,784 | 14,438,884 | 12,661,474 (87.8%) |
|  | Stressed | 1 | 13,734,523 | 13,687,826 | 11,939,870 (87.2%) |
|  |  | 2 | 14,612,044 | 14,565,285 | 12,929,069 (88.7%) |
|  |  | 3 | 15,216,799 | 15,158,975 | 13,349,636 (88.0%) |
| Total |  |  | 263,364,410 | 262,427,357 | 229,863,563 (87.6%) |
