# Supplementary figures and images for: Time-course transcriptomic analysis of Petunia ×hybrida leaves under water deficit stress using RNA sequencing
Source: PLoS One. 2021 Apr 26;16(4):e0250284. doi: 10.1371/journal.pone.0250284 (PMC8075263; doi:10.1371/journal.pone.0250284)

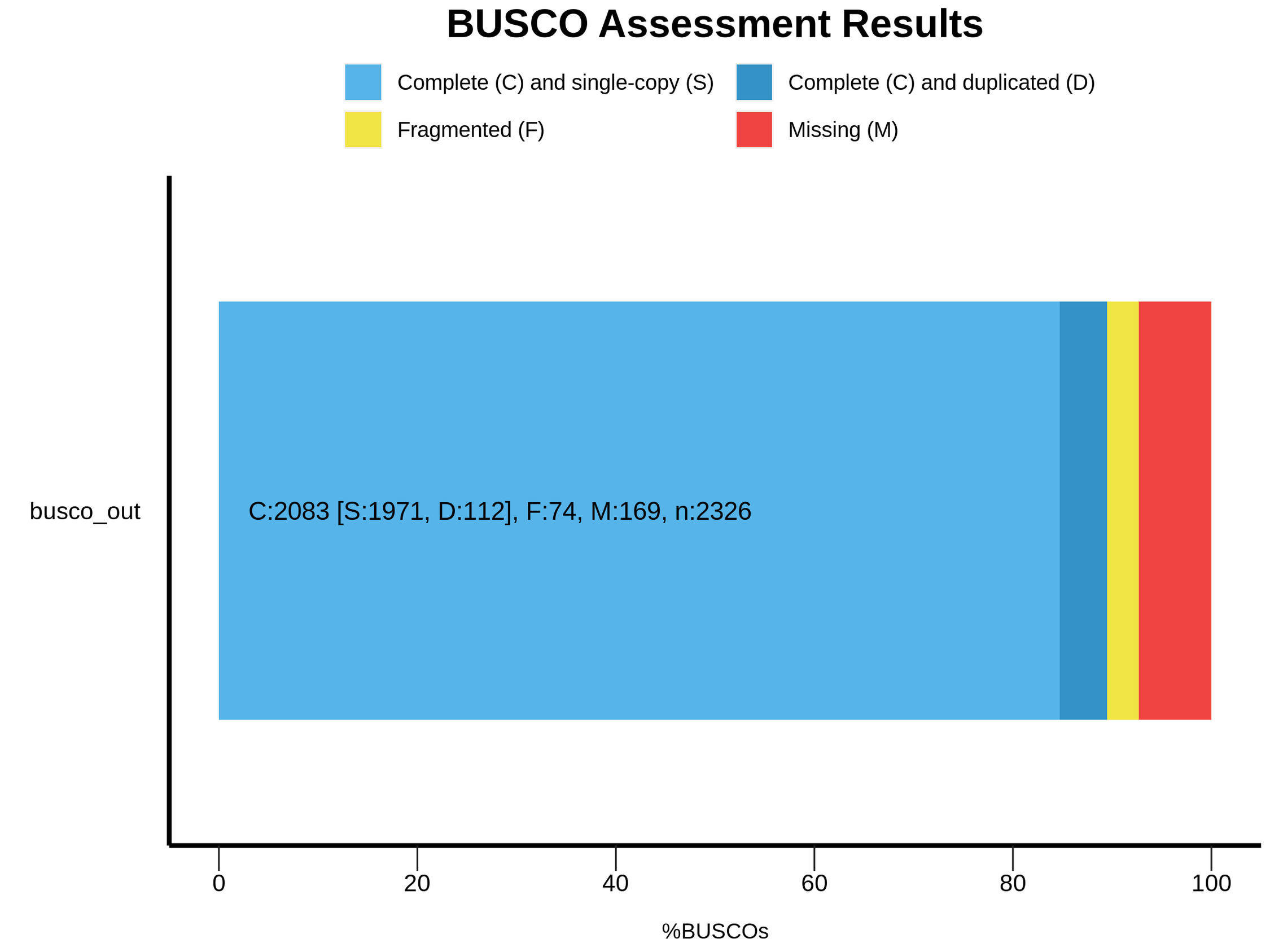

Supplement: S1 Fig — (TIF) [file pone.0250284.s003.tif]

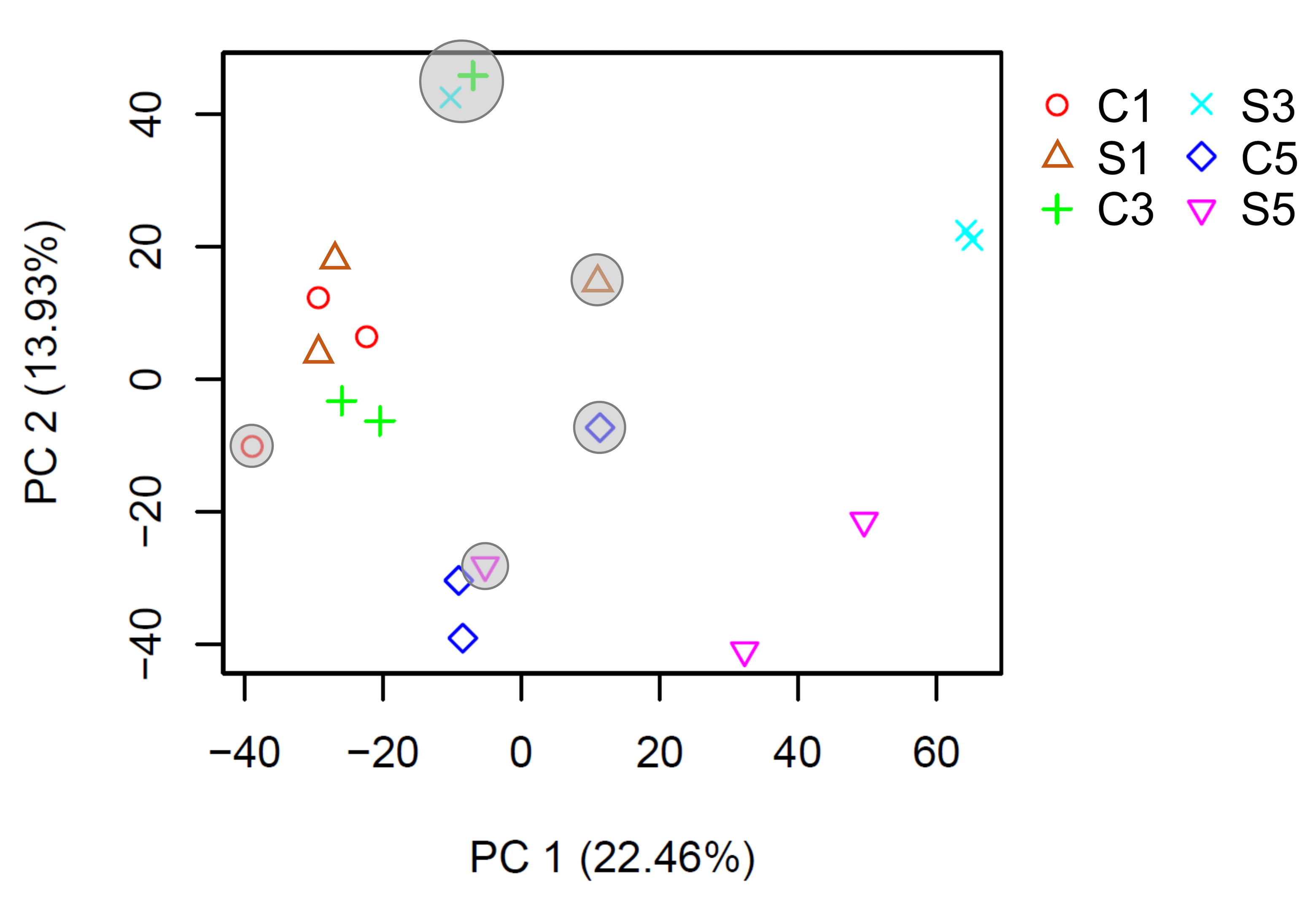

Supplement: S2 Fig — C and S correspond to control and stressed plants, respectively, and the numbers indicate the number of days after withholding water. Gray-circled samples are considered as outliers, and they were excluded for further analyses. (TIF) [file pone.0250284.s004.tif]

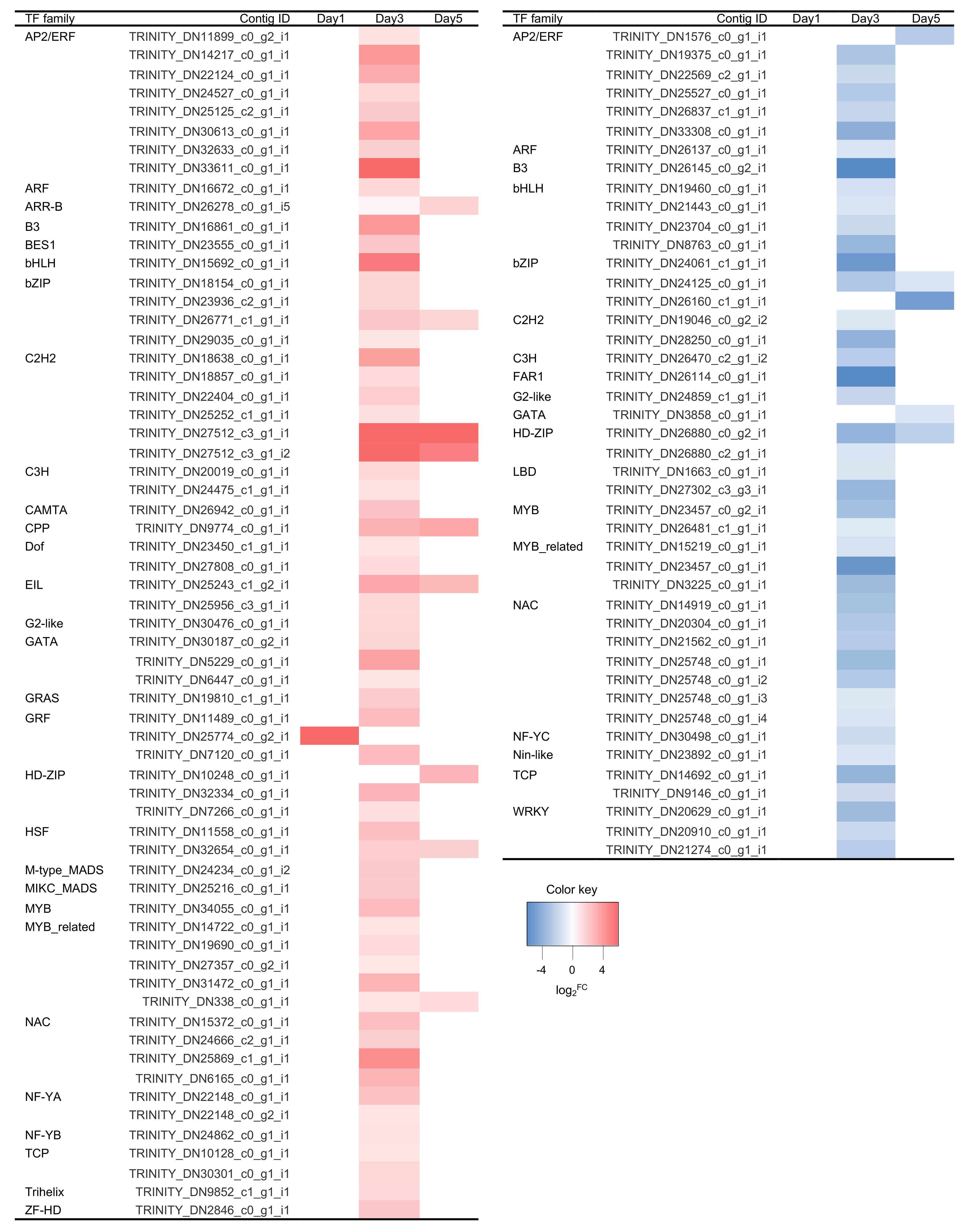

Supplement: S3 Fig — Upregulated (left and pink) and downregulated (right and blue) TFs. (TIF) [file pone.0250284.s006.tif]
